# Minimum acceptable diet and associated factors among 6–23 months old children enrolled in outpatient therapeutic program in the Tulla district, Sidama region, Ethiopia: a community-based cross-sectional study

**DOI:** 10.1186/s41043-024-00581-9

**Published:** 2024-07-08

**Authors:** Mesfin Markos, Beniyam Samuel, Alemzewed Challa

**Affiliations:** 1 Health office, Tulla District, Sidaama Region, Hawassa, Ethiopia; 2 Department of Midwifery, College of Medicine and Health Science, Dilla University, Dilla, Ethiopia; 3 School of Nutrition, Food Science and Technology, College of Agriculture, Hawassa University, Hawassa, Ethiopia

**Keywords:** Knowledge, Complementary feeding, Minimum acceptable diet, Outpatient therapeutic program, 6–23 months old children, Ethiopia

## Abstract

**Background:**

Improving the minimum acceptable diet (MAD) is essential for ensuring optimal growth and development of children, as well as preventing malnutrition and its consequences. Previous studies in Ethiopia have focused on the magnitude and determinants of a minimum acceptable diet. However, much emphasis was not given to minimum acceptable diet and its associated factors among 6–23 months old children enrolled in Outpatient therapeutic programs (OTP), particularly, in the study area. This study determines the minimum acceptable diet and associated factors among 6–23-month-old children enrolled in OTP.

**Methods:**

A community-based cross-sectional study was conducted among 346 randomly selected mothers with children aged 6–23 months who were admitted to the OTP. The data were collected using interviewer-administered structured questionnaires. The data were entered, cleaned, coded into Epidata version 4.6, and exported to SPSS version 26 for further analysis. Multivariate logistic regression was used to assess the determinants of MAD.

**Results:**

The overall prevalence of minimum Acceptable diet among children aged 6–23 months enrolled to OTP was 14.5% (95% CI: 12.02-19%). The odds of MAD were 1.9 times higher among children aged 18–23 months compared to children aged 6–11 months (AOR = 1.9, 95% CI ((1.2 3.9). The odds of MAD were 2.9times higher in children whose mothers had a good knowledge on recommended feeding (AOR = 2.9, 95% CI (1.2, 6.35). Mothers who had no formal education were 81% less likely to provide minimum acceptable diets for their children compared to their counterpart.(AOR = 1.94, 95% CI = 1.24, 4.19).

**Conclusion:**

The practice of a minimum acceptable diet is inadequate. Nutrition education should be emphasized to improve the mothers’ nutrition knowledge regarding infant and young child feeding recommendations, to support mothers in overcoming barriers to feeding their children with adequate diets, and to foster complementary feeding practices for malnourished children.

## Introduction

A child’s minimum acceptable diet (MAD) is the percentage of 6-to 23-month-olds who had a minimum diverse diet and minimum frequency of meals the day before [1, 2]. For children between the ages of 6 and 23 months, appropriate feeding practices are essential because this is the ideal time for their growth and development [3]. Given their increased vulnerability to illness, mortality, and malnourishment, children under the age of two benefit greatly from varied diets and regular meal schedules [4]. The minimum acceptable diet (MAD) is useful for inadequate nutrient intake and is one of the most significant indicators for assessing infant and young child feeding (IYCF) practices. MAD is among eight main indicators the World Health Organization (WHO) developed in 2007 to evaluate infant and young child feeding (IYCF) practices for children aged 6 to 23 months [5] It combines standards of dietary diversity and feeding frequency based on feeding status [6, 7]. Minimum Dietary Diversity (MDD) focuses on the variety of food groups consumed by children. Children should receive foods from different food groups to ensure adequate nutrient intake. Meeting MDD means that a child consumes foods from at least four out of seven food groups within 24 h. Minimum Meal Frequency(MMF) emphasizes the frequency of feeding and ensures that children receive sufficient meals throughout the day, and recommended meal frequency varies by age; 6–8 months child should take 2–3 meals per day, plus breast milk, and 9–23 months child should take 3–4 meals per day, plus breast milk. Poor dietary practices, including inadequate MAD, MDD, and minimum meal frequency, contribute to undernutrition [5, 8].

Undernutrition, especially during the initial two years of life, is associated with elevated rates of illness and death, along with inadequate brain growth, which in turn affects adult cognitive development, intellectual capacity, and economic productivity [9, 10]. Based on UNICEF, In the year 2019, 47 million children under the age of five were wasted at all times, with 75% of them residing in lower- and middle-income nations [11].

There are many possible causes for low minimum acceptable diet (MAD) among children aged 6–23 months, depending on the context and factors that influence child feeding practices. Some examples include low education, lack of knowledge, poor health, and cultural beliefs, which affect the quality and quantity of complementary foods offered to children. Household and environmental factors, such as poor sanitation, hygiene, and water quality, increase the risk of infections and malabsorption of nutrients in children [12].

Globally, in 2020, the prevalence of MAD was 16.6% among children aged 6–23 months [13]. Few children receive adequate and safe complementary foods; in many countries less than a fourth of infants 6–23 months of age meet the criteria of MDD and minimum meal frequency that are appropriate for their age. It was estimated that 149 million children under the age of five were stunted (too short for their age) and s45 million wasted (too little for their height) [14]. The regions of Africa and South-East Asia have the highest rates of undernutrition; where the former is responsible for about 39.4% of stunted, 24.9% of underweight, and 10.3% of wasted children under the age of five [15]. Based on a meta-analysis conducted in 32 countries in sub-Saharan Africa, few children received nutritionally adequate and safe complementary foods. In many countries, less than a quarter of infants aged 6–23 months meet the criteria of dietary diversity and feeding frequency that are appropriate for their age, and the prevalence of malnutrition is highest in East and West Africa [16]. According to the study conducted in Ghana, Nigeria, Uganda, and Kenya, only 29.9%, 8⋅36%, 7⋅3%, 23.9%, and 48.5% of children obtained the recommended minimum acceptable diet, respectively [17–20]. A study conducted in Ethiopia showed that only a small proportion of children received minimum dietary diversity and maternal knowledge of complementary feeding practices was low [21–24]. The magnitude of MAD varies from 7 to 74% in Ethiopia [25–27]. According to the findings of the 2019 Ethiopian Demographic Health Survey(EDHS), only 11.3% of Ethiopian young children were found to have consumed the Minimum Acceptable Diet [28].

Mothers’ education age, and knowledge, fathers’ income, age and gender of the child, wealth status, marital status, and the presence of a family member who fed the child without fasting, postnatal visit, and media exposure were significantly associated with MAD [8, 29].

The Ethiopian government has planned different nutrition-related targets to end child malnutrition by 2030 by implementing different programs and strategies in its development plans such as the Ethiopian Food and Nutrition Policy [30], the Seqota Declaration [31], the second National Nutrition Program(NNPII) [32], the second Health Sector Transformation Plan II (HSTPII) [33], and Sustainable Undernutrition Reduction(SURE) [34]. Determining the prevalence and determinants of MAD helps to obtain evidence-based information based on the study’s area context, and monitor, and evaluate the impact of nutrition programs and policies on the dietary quality, diversity, and acceptable diet of young children. In Ethiopia, Even though studies were conducted on minimum acceptable diet and its associated factors among 6–23 months children in Ethiopia, there are limited information among 6–23 months children who enrolled in outpatient therapeutic programs, particularly in the study area. Therefore, this study aimed to determine a minimum acceptable diet and associated factors among 6–23 months children who enrolled in outpatient therapeutic programs.

## Methods and materials

### Study design, study setting, design, and participants

A community-based cross-sectional study was conducted from October 5 to November 30, 2023, in the Tulla District, Sidama Region of Ethiopia. The district has a population of approximately 248,710 people, with 23,199 children under five years old. The district has one primary hospital, six health centers, four private clinics, and 12 drug outlets. The local community relies on farming and growing seeds of maize, chat, and coffee. This study focused on mothers of 6-23-month-old children who were enrolled in the outpatienttherapeutic program(OTP). The source population consisted of all mothers and carers enrolled in the OTP in the Tulla District administration, with children between the ages of 6 and 23 months. The study population included mothers and carers of children with mild acute malnutrition, aged 6 to 23 months, who were enrolled in the OTP and lived in randomly selected kebeles. Individual mothers or carers who had children between the ages of six and twenty-three months resided in the randomly chosen Kebeles, enrolled in the OTP, and took part in the actual data collection were the study units. Mothers with mild acute malnutrition, whose 6- to 23-month-old infants were included in the Tulla district’s Outpatient Treatment Programme (OTP), were included in the study. Mothers with children with severe acute malnutrition who also had heart illness, vomiting, or grade two or three nutritional edoema were excluded from the study. The sample size was determined using a single population proportion formula by considering the proportion of MAD in Mareka District, southern Ethiopia, which was 35% [23, 27], with a 5% margin of error and a 95% confidence level. The calculated sample size was 352. The final sample size was 370, after using a 5% nonresponse rate. A list of mothers with children aged 6–23 months enrolled in an OTP program was obtained from the health bureau of Tulla District. Children’s dates of birth and the number of houses were also recorded. The list was based on the community health campaign conducted by trained health extension workers in all 12 Kebeles of Tulla Woreda in August 2023. Children with MAM (MUAC: 11.5 × 12.5 cm, no medical complications, and bilateral pitting edema) were registered separately. There are 12 Kebeles in the Tulla District administration, out of which six kebeles (with an estimated 1315 study participants) were randomly selected using a lottery method. To provide an equal chance in the selection, a proportional allocation technique was employed across each selected kebele. A systematic random sampling technique was used to select 370 participants.

### Data collection tools

We adapted the Food and Agriculture Organization(FAO’s) questionnaires for measuring nutrition and feeding knowledge, attitudes, and practices of infants and young children [35]. The knowledge scale consisted of seven questions, some open-ended and some multiple-choice, which tested participants’ understanding of the recommendations. Each question was scored one point for a correct answer, and total scores were generated for each participant and computed out of 100%. The attitude scale consists of seven items that use a 3-point Likert. Two forms of the 3 likert scale were used depending on whether the item was assessing perceived barriers or perceived benefits. For preceived barriers the responses were: 1- not difficult, 2- So-so, and 3-Difficult. For perceived barriers: 1-not good, not sure, and 3-Good. In order to ensure higher scores denoted poetive attitude, items for the perceived barriers were reversed score(i.e. 1 = 3, 2 = 2, 3 = 1). Total scores were generated for each participant and computed out of 100%.

To assess the variety of foods consumed by children, we used a list of seven food groups and asked mothers to mark the foods consumed by their children in the last 24 h. We followed the Food and Agriculture Organization (FAO)guidelines to count the number of different food groups consumed by each child. We used the mothers’ answers to calculate three indicators of complementary feeding: minimum meal frequency, MDD, and MAD.

### Operational definitions

#### Minimum meal frequency

The number of times a child aged 6–23 months has received complementary foods in the last 24 h [6].

#### Minimum dietary variety

The number of food groups (out of seven) that a child consumed in the last 24 h).

#### Minimum acceptable diet

The percentage of children who met both minimum meal frequency and minimum dietary diversity [6].

### Data and quality assurance

The data collectors used interviewer-administered questionnaires, an OTP chart, and Logobook to collect data. Study participants were asked about their socio-demographic characteristics, knowledge, attitude, and practice of complementary feeding(CF). The questionnaires prepared in the English language were translated into the local language (Sidaamu Afoo) and retranslated back into English to check the consistency of their meaning. The questions were tested for accuracy and clarity before the study in a nearby town, Woreda, Hawella, with a sample size of 5%. Pre-testing also helped estimate the time required for the interviews. The questions were revised based on feedback and interpretation consistency. The supervisor reviewed the questionnaires daily to ensure uniformity, completeness, consistency, and missing data. The principal investigator held a brief meeting with the supervisor each day to verify the correct data collection before it was completed.

### Statistical analysis

The data were entered, cleaned, coded into Epidata version 4.6, and transferred to SPSS version 25 for further analysis. Descriptive statistics such as frequencies, percentages, and interquartile ranges were computed. Bivariate and multivariate logistic regression analysis were conducted to determine the degree of association between outcome and predictor variables. Independent variables with a P-value of less than 0.25 during bivariable analysis were selected for multivariable logistic regression analysis. A multivariate logistic regression analysis was performed to identify the statistically significant variables. Statistical significance was set at a P-value of less than 0.05, and model fitness was checked using Hosmer and Lemeshow’s goodness of fit. The variance inflation factor (VIF) was used to evaluate the potential for multicollinearity among independent variables.

## Result

### Socio-demographic characteristics of children caregiver’s

The study involved 346 caregivers of children on the OTP out of the planned 370 (93.5% response rate). Almost all the mothers of the children were caregivers (99.1%). Most children were 18–23 months old (54.6%). The participants were mostly Protestants (81%), married (98.8%), or unemployed (80%). More than half had no formal education (54.3%)(Table 1).

#### Caregivers’ knowledge on complementary feeding

Most mothers and caregivers (71.7%) knew how long they breastfed their babies. They also knew the right time to start giving complementary foods after 6 months (76%). However, they did not explain why this was important or how to make food more nutritious and diverse. Only 224 (64.7%) and 62.1%) knew these aspects, rspectively. Of total study pariticipants, **209** (60.5%) had good knowle dge of the recommended feeding practices(Table 2).

#### Caregivers’ attitude on complementary feeding

Most mothers (88.6%) felt confident about making food for their children, but nearly half (46.8%) had trouble with food variety. Almost all mothers (98%) understood the benefits of breastfeeding for the past 6 months, but some (13.3%) faced challenges in doing so. the majority of mothers (57.5%) had a posetive attitude towards the feeding recommendation (Table 3).

#### Magnitude of minimum adequate diet

A summary of the food intake of the children in the last day is as follows: Out of 346 children, only 55% ate legumes and nuts, 60% ate grains, roots, and tubers, 55% ate dairy products, 20.3% ate flesh foods, 23. % ate eggs, 28% ate vitamin A-rich foods, and 6.4% ate fruits and vegetables. Most of the mothers (96.9%) were still breastfeeding their children. Only 13% of the children had a diverse diet, 39.5% had a frequent diet, and 14.5% had an adequate diet.

#### Factors associated with minimum acceptabele diet

In the binary logistic regression, the monthly income greater than 1,000 Ethiopian Birr, mothers who do not have formal education, children who were aged 12–17 and 18–23 months, a child’s father earning adequate income for family upkeep, knowledge level, and positive attitude were factors associated with the minimum adequate diet. However, mothers who do not have formal education, mothers whose knowledge level is greater than 70%, and children who were aged 18–23 months were significantly associated factors in the multivariable logistic regression model. The odds of an adequate diet were 1.9 times higher among children who were aged 18–23 months compared to children who were aged 6–11 months (AOR = 1.9, 95% CI (1.2 to 3.9). The odds of an adequate diet were 2.9 times higher in children whose caregiver had greater than 70% knowledge of recommended feeding (AOR = 2.9, 95% CI (1.2, 6.35). The odds of an adequate diet for the children were 81% less likely in a caregiver who did not attend formal education compared to their counterparts (AOR = 1.94, 95% CI = 1.24, 4.19)(Table 4).

**Table 1:** Socio-economic characteristics of caregivers and demographic characteristics of caregivers of children 6–23 month of age admitted at OTP in Tulla district, Sidaama region, Ethiopia, 2023 (*N* = 346)

Variables	Response category	Frequency	Percent
Age of children in years	6–11 Months	38	11
	12–17 Months	119	34.4
	18–23 Months	189	54.6
Sex of Child	Male	167	48.3
	Female	179	51.7
Children MUAC during OTP entry	$\le$10 cm	220	64
	$\le$11.5 cm	126	36
Weight of children after OTP entry	$\le$5kg	72	20.8
	$\le$8kg	274	79.2
Marital status	Married	34 2	98.8
	Single	4	1.2
Education of mothers/ caregivers	No formal education	188	54.3
	Formal education	158	45.7
Family size	≤ 4	137	39.5
	> 4	209	60.5
Number of < 5children	One	60	17.3
	Two and above	286	82.7
Religion of Caregiver’s	Protestant	281	81
	Orthodox	26	7.5
	Muslim	40	11.5
Employment Status of the mother	Employed	277	80
	Unemployed	68	20
Age of caregiver	**15–17**	2	0.6
	17–19	13	3.8
	19–21	31	8.9
	21–30	206	59.5
	> 30	94	27.2
Children primary caregiver	Mother	343	99.1%
	Father	3	0.9%
Monthly income	< 500Birr	221	64
	500-1,000 Birr	87	25
	> 1,000 Birr^+^	38	11
Child’s father earns adequate income for family upkeep	Yes	202	58.3
	No n	144	41.7

**Table 2:** Complementary feeding knowledge among caregivers of children 6–23 month of age admitted at OTP in Tulla District, Sidaama Region, Ethiopia, 2023 (*N* = 346)

	Category	Frequency	Percent
Correctly answered recommended duration of continued breastfeeding	Yes	248	(71.7%)
	No	98	(28.3%)
Correctly answered age of start of complementary foods	Yes	263	(76%)
	No	83	(24%)
Gave good reasons for giving complementary foods at 6 months	Yes	122	(35.3%)
	No	224	(64.7%)
Correctly knew how to ensure consistency of meal	Yes	211	(61%)
	No	135	(39%)
Gave good reasons why consistency of meal of meal is necessary	Yes	169	(48.8%)
	No	177	(51.2%)
Correctly knew how to ensure dietary diversity and ways of enriching porridge	Yes	131	(37.9%)
	No	215	(62.1%)
Knew responsive feeding	Yes	251	(72.5%)
	No	95	(27.5%)
Classification of knowledge scores	High (> 70%)	209	(60.4%)
	Low	137	(39.6%)

**Table 3:** Complementary feeding attitude among care-givers of children 6–23 months of age admitted at OTP in Tulla District, Sidaama Region, Ethiopia, 2023 (*N* = 346)

Characteristics	Category	Frequency	Percent
Feels confident in preparing food for child	Yes	306	(88.6%)
	No	40	(11.4%)
Perceives that giving different types of food is beneficial to child	Yes	238	(68.8%)
	No	108	31.2%)
Has difficulty giving different types of food to child	Yes	162	(46.8%)
	No	184	(53.1%)
Perceives that feeding child several times each day is beneficial	Yes	295	(85%)
	No	51	(15%)
Has difficulty feeding child seve ral times a day	Yes	139	(40.2%)
	No	207	(59.8%)
Perceives that its beneficial to continue breastfeeding beyond 6 months	Yes	339	(98%)
	No	7	(2%)
Has difficulty continuing to breastfeeding beyond 6 months	Yes	46	(13.3%)
	No	300	(86.7%)
Attitude classification	Positive(> 70%)	199	(57.5%)
	Less Positive	147	(42.5%)

**Table 4:** Multivariable Logistic regression analysis of factors associated with minimum acceptable diet among care-givers of children 6–23 months of age admitted at OTP in Tulla District, Sidaama Region, Ethiopia,, 2023 (*N* = 346)

Variables	MAD				
	Yes (%)	No (%)	(COR) (95% CI)	*p*-value	(AOR) (95% CI)
Educational level					
No formal education	11(6.3%)	178(93.7%)	0.2(0.290–1.080)	0.03	0.19(0.095–0.456)**
Formal education	36(21.8%)	121(78%)	1.00	0.06	1.00
Age of children in years					
6–11 Month	8(21%)	30(79%)	1.00	0.85	1.00
12–17 Month	19(14%)	99(86%)	1.4(1.02, 3.3)	0.67	1.2 (0.70, 3.42)
18–23 Month	20(15%)	170(85%)	2.2 (1.4, 4.5)	0.04	1.9 (1.2 to 3.9) *
Monthly income					
< 500Birr	9(4.3)	214(95.7)	1.00	0.46	1.00
500-1,000 Birr	9 [7]	77(93)	0.36(0.28, 1.12)	0.69	0.46(0.41,1.02)
> 1,000 Birr^+^	29(81)	8 [19]	0.01(0.012, 0.08)	0.64	0.021(0.01, 1.01)
knowledge level					
High knowledge level	38 [19]	171(81)	3.2(1.7, 5.94)	0.019	2.9(1.2, 6.35)*
Low	9 [8]	128(92)	1.00	0.32	1.00
Attitude					
Posetive attitude(> 70%)	32 [14]	166(86)	1.7(1.04, 4.73)	0.347	1 0.53 (0.90, 3.54)
Less posetive attitude	15 [15]	133 (85)	1.00	0.87	1.00
Child’s father income					
Yes	32 [19]	169(81)	1.6(1.10,3.49)	0.78	1.5(0.82, 4.19)
No	15(10.7)	130(89.3)	1.00	0.94	1.00

*Statistical significant at *P* < 0.05, ** at *P* < 0.01 and *** at *P* < 0.001

## Discussion

A minimum acceptable diet (MAD) is a measure of the quality and quantity of complementary feeding for children aged 6–23 months. It is based on the combination of dietary diversity and meal frequency. Conducting a study on MAD can help to assess the nutritional status and feeding practices of young children, identify the factors that influence their diet, and design interventions to improve their health and development. This study aimed to determine a minimum acceptable diet and associated factors among 6–23 months children who enrolled at outpatient therapeutic programs in Tulla District, Sidama Region, Ethiopia.

This study showed that only 14.5% (95% CI: 12.02-19%) of the children aged 6–23 months who were enrolled in the outpatient therapeutic program met the minimum adequate diet criteria. This suggests that the complementary feeding practices in the study area were inadequate and could lead to have several deterimental effect upon young children. Therefore, it is important to implement effective interventions to improve the nutritional status of these children. The quality and quantity of food that a child receives in the first years of life can have a lasting impact on their health and development. Feeding practices that are unsuitable or inadequate can increase the child’s vulnerability to malnutrition, vitamin deficiencies, diarrhea, and respiratory tract infections. On the other hand, feeding the child with appropriate and sufficient food can enhance their mental and motor development, reduce their risk of obesity, protect them from various infectious diseases and their mortality, and improve their overall development [36–38]. This study finding is higher than studies conducted in Tigray (2.30%) [39], North West, Ethiopia (12.6%) [1], EDHS 2016(6.10%) [40], Uganda (5.34%) [41], India(8·4%) [42], Dembecha (8.60%) [26]. However, lower than the studies conducted in Addis Abeba(76.6%) [25], Mareka District(35.5%) [27], Bangladesh(38%) [43], Lalibela, northeast Ethiopia(16.7%) [44], Myanmar (16.00%) [45], Delhi (19.70%) [46], Congo(33%) [47], central Amhara (31.60%) [48], Kaski (42.40%) [49], Abu Dhabi(36.20%) [50], Ghana (24.90%) [50], Rural Madagascar (50%) [51], and Bangladesh (23.00%) [52]. The possible reason for the discrepancy in the findings could be the fact that the studies’ contexts, economic backgrounds of the care givers, and data collection seasons variations. Furthermore, variations in study methodology, sample size, study period, and sociodemographic variation may account for the level of discrepancies between the findings.

Our study shows that most mothers(76%) knew the right time to start giving other foods besides breast milk, but the majority of mothers (64.7%) did not explain why they should do so at six months. It also shows that less than half of the mothers (37.9%) knew how to make the food more nutritious and varied. These knowledge gaps could be dangerous because they could lead to early or late introduction of complementary foods, which could affect the child’s growth and development negatively. Therefore, this suggests that health workers should give more counseling and education on nutrition during the regular sessions of growth monitoring and childcare events.

We assessed the mothers’ attitudes toward feeding habits for infants and young children in this study, as these practices are critical to the growth and development of children. While most women (85%) believe that feeding their children multiple times a day is good, over 40.2% of them said they found it challenging to feed their children multiple times a day. While the majority of women feel comfortable making meals for their children, nearly half of them said they had trouble doing it. These challenges may have contributed to the low degree of meal frequency and dietary diversity that we found in our study, which may have had detrimental effects on the children’s health and nutritional status. The mothers’ low income may be a contributing factor in these issues since it may restrict their access to a variety of wholesome foods. In reality, the majority of the mothers claimed that their inability to provide their children with a variety of meals at the suggested times was due to their lack of income. The economic barriers that mothers face should also be addressed by interventions aimed at improving infant and young child feeding practices. Healthcare providers should also offer individualized counseling to mothers and other caregivers during child healthcare sessions to help them identify the unique obstacles they face in adhering to the recommendations for infant and young child feeding. Mothers and other caregivers may receive encouragement to implement the best feeding habits during these sessions, as well as support in overcoming some of the challenges they encounter. This technique will promote children’s growth and health, as well as the amount and quality of feeding practices for infants and early children.

In this study, several factors were identified that are associated with a minimum acceptable diet among 6–23-month-old children who were admitted to OTP. Mothers who did not have formal education, mothers who had a good knowledge of young children’s feeding practices, and children who were aged 18–23 months were significantly associated with the minimum acceptable diet.

Based on this study, mothers who had no formal education were 81% less likely to provide minimum acceptable diets for their children compared to mothers who had no formal education. This finding was supported by studies done in Dembecha and Goncha districts, in northwest Ethiopia, respectively [1, 26]. These studies reported that mothers who had formal education were more likely to provide the minimum acceptable diet for their children compared to mothers who had no formal education. This could indicate that education helps mothers understand the advantages of child-feeding practices and contributes to achieving a minimum acceptable diet.

Our study showed that the odds of an adequate diet were 1.9 times higher among children who were aged 18–23 months compared to children who were aged 6–11 months (AOR = 1.9, 95% CI (1.2 to 3.9). This finding was supported by a previous study that was conducted in Debre Berhan Town, Ethiopia [48]. This finding was supported by a previous study that was conducted in Debre Berhan Town, Ethiopia [48]. The possible reason could be the mothers’ perceptions of the inability of their young children’s stomachs to break down solid or semisolid food at an early age and the benefits of introducing a varied solid and semisolid diet instead of just a milk-based feeding after the infant turns 12 months old.

A minimum adequate diet(MAD) is essential for the growth and development of children. It means that the children receive a variety of foods from different food groups and that they meet the minimum requirements for frequency and quantity of feeding. One factor that influences MAD is the mother’s knowledge of infant and young child feeding recommendations. This study showed that children whose mothers had a good knowledge of these recommendations were 2.9 times more likely to have a minimum adequate diet than those whose mothers did not. This result is consistent with a previous study in Ghana [29], which also found a positive association between a mother’s knowledge and a child’s diet. The reason for this may be that mothers who are well-informed about infant and young child feeding recommendations try to ensure and provide a minimum acceptable diet for their children.

### Strengths and limitations

The current study had several limitations and strengths. One strength of our study is that we designed a community-based study. However, the utilization of the cross-sectional study design method has limitations due to its inability to identify causality. Recall bias might also need consideration when interpreting our findings. Notwithstanding this, the study has important strengths. The findings broaden our knowledge of the prevalence of feeding practices for infants and young children as well as the factors that contribute to them by adding to the body of existing research on the subject. The results open up new directions for designing interventions to enhance the eating patterns of infants and young children in the study area area and throughout Ethiopia.

### Conclusion and recommendation

The magnitude of a minimum acceptable diet is inadequate in study area. Mothers who did not have formal education, mothers who had a good knowledge of recommended feeding, and children who were aged 18–23 months were significantly associated with a minimum adequate diet. Nutritional education through multi-media campaigns, education of mothers and caregivers on nutrition, promotion of breastfeeding, and increasing production of local complementary foods have a vital role in ensuring an adequate diet and increasing maternal knowledge on complementary feeding among children aged 6–23 and admitted for outpatient treatment of moderate malnutrition.

## Data Availability

On reasonable request, the corresponding author will provide the complete data set and additional study-related information.

## Abbreviations

CF: Complementary feeding
CI: Confidence interval
EDHS: Ethiopian Demographic survey
MAD: Miniumum acceptable diet
CHD: Community health day
MAUC: Middle uppper arm curcumfrance
OTP: Out patient program
MAM: Miniumum acceptable meal
MMF: Miniumum meal frequency
MDD: Miniumum diet diversity
